# Propanol and 1, 3-propanediol enhance fatty acid accumulation synergistically in *Schizochytrium* ATCC 20888

**DOI:** 10.3389/fmicb.2022.1106265

**Published:** 2023-02-09

**Authors:** Tiantian Wang, Fangzhong Wang, Lei Zeng, Pengfei Guo, Yawei Wu, Lei Chen, Weiwen Zhang

**Affiliations:** ^1^Laboratory of Synthetic Microbiology, School of Chemical Engineering and Technology, Tianjin University, Tianjin, China; ^2^Frontier Science Center for Synthetic Biology, Key Laboratory of Systems Bioengineering (Ministry of Education), Tianjin University, Tianjin, China; ^3^Center for Biosafety Research and Strategy, Tianjin University, Tianjin, China

**Keywords:** propanol, 1, 3-propanediol, fatty acid profile, GC–MS based metabolomics, *Schizochytrium* ATCC 20888

## Abstract

The effects of propanol and 1, 3-propanediol on fatty acid and biomass accumulation in *Schizochytrium* ATCC 20888 were explored. Propanol increased the contents of saturated fatty acids and total fatty acids by 55.4 and15.3%, while 1, 3-propanediol elevated the polyunsaturated fatty acids, total fatty acids and biomass contents by 30.7, 17.0, and 6.89%. Although both of them quench ROS to increase fatty acids biosynthesis, the mechanisms are different. The effect of propanol did not reflect on metabolic level while 1, 3-propanediol elevated osmoregulators contents and activated triacylglycerol biosynthetic pathway. The triacylglycerol content and the ratio of polyunsaturated fatty acids to saturated fatty acids were significantly increased by 2.53-fold, which explained the higher PUFA accumulation in *Schizochytrium* after adding 1, 3- propanediol. At last, the combination of propanol and 1, 3-propanediol further elevated total fatty acids by approximately 1.2-fold without compromising cell growth. These findings are valuable for scale-up production of designed *Schizochytrium* oil for various application purposes.

## 1. Introduction

Fatty acids can be classified into two major categories of saturated fatty acids and unsaturated fatty acids. Saturated fatty acids have been commonly used to provide energy and cholesterol for the human body (Mensink et al., 2016) and also the main component of renewable biodiesel. Unsaturated fatty acids can be further divided into monounsaturated and polyunsaturated fatty acids based on their different number of double bonds. Polyunsaturated fatty acids (PUFAs), of which the carbon chain length is typically 18 to 22 carbon atoms, includes eicosapentaenoic acid (EPA), docosapentaenoic acid (DPA) and docosahexaenoic acid (DHA). EPA, known as blood vessel scavenger, can prevent atherosclerosis by cleaning up cholesterol and triglycerides in human blood vessels (Adarme-Vega et al., 2014); DPA plays an important role in repairing damaged blood vessels, fighting against chronic inflammation, improving lipid metabolism, and promoting the absorption of EPA and DHA (Miller et al., 2013); DHA, the main component of human retina and brains, has an important impact on fetal neural development, infant cognitive development and vision (Mallick et al., 2019; Martins et al., 2020).

The traditional sources of fatty acids include plants, animals and microorganisms (Diao et al., 2020) however, most of these approaches carry issues of the high cost and environmental pollution, as well as low sustainability. In recent years fatty acids produced by microalgae has received significant attention (Barbosa, 2010). Among them, *Schizochytrium* is a eukaryotic heterotrophic microalgae with the ability to accumulate high-level polyunsaturated fatty acids, with the DHA content reaching up to ~20% of dry cell weight (Chang et al., 2013). It is thus considered as an important alternative to fish oil for industrial production of fatty acids, also because the DHA is high purity and without noisy fish smell (Ryckebosch et al., 2014).

At present, the approach to improve fatty acids content in *Schizochytrium* mainly include laboratory adaptive domestication (Sun et al., 2016; Hu et al., 2021), mutagenesis (Zeng et al., 2021), genetic engineering approach (Ren et al., 2018) and regulation by chemical regulators (Zhang et al., 2016; Concha et al., 2018). Adaptive domestication relies on natural mutation and external pressure to screen strains of high productivity, which takes a relatively long time. Mutagenesis is random, and the obtained desired phenotypic mutants need high-throughput screening, leading to high cost. The genetic transformation system is relatively immature for *Schizochytrium*, leading to difficulty to effectively improve fatty acid content through metabolic engineering method. In contrast, regulation by chemical modulators is a useful alternative strategy to improve fatty acid accumulation in large-scale cultivation of microalgae (Yu et al., 2015; Sun et al., 2019; Du et al., 2021). Some chemicals have been demonstrated to have a significant impact on cell growth and lipid accumulation in microalgae. For example, the addition of methanol, ethanol and butanol were found to increase the content of total fatty acids, change the fatty acid profile, and promote the synthesis of saturated fatty acids in *S*. *limacinum* B4D1 (Zhang et al., 2017; Du et al., 2019; Zhu et al., 2022); however, due to impairing growth, the total yield of fatty acid was decreased in the studies. Therefore, searching for chemical modulators that can enhance fatty acid productivity without compromising cell growth in *Schizochytrium* is necessary.

Propanol is a colorless transparent liquid generally used as a solvent. Previously, there was report that propanol was used as an additive to improve the content of fatty acids in *Rhodococcus opacus* PD630 (Zhang et al., 2019). Compared with propanol, 1, 3-propanediol (1,3-PD) has an additional hydroxyl group at the last carbon atom, and its effects on microbial metabolism could be different from those of propanol. Propanol can also be utilized as carbon source to enhance the biosynthesis of propionyl-CoA, the precursors of odd fatty acids (Zhang et al., 2019), while 1, 3-propanediol can only be degraded by strictly anaerobic gram-negative bacteria, and it might therefore play a role in osmoregulation in most microbes (Oppenberg and Schink, 1990; Wang et al., 2015). However, there is no report on the regulation of propanol and 1,3-PD in the microalgal cultivation.

In this study, two chemical additives, propanol or 1,3-PD, was, respectively, added into the cultures of *Schizochytrium* ATCC 20888 during shaking flask fermentation process to explore their effects on biomass accumulation and fatty acid synthesis. The GC–MS based metabolomics were then applied to determine the metabolic changes in response to the treatments. At last, the two additives were simultaneously added to test the synergistic effect on biomass and fatty acid in *Schizochytrium* ATCC 20888. Based on these results, a new strategy to modify fatty acid profiles in *Schizochytrium* ATCC 20888 was proposed. These findings are valuable for industrial application of *Schizochytrium* for high-content fatty acid accumulation.

## 2. Materials and methods

### 2.1. Strain and chemicals

*Schizochytrium* ATCC 20888 was purchased from the American Type Culture Collection (MD, USA), stored in a tube containing 30% (*v*/*v*) glycerol concentration and frozen at −80°C. Fatty acid standards were purchased from Sigma-Aldrich (St. Louis, MO, United States). Yeast extract was purchased from OXOID (Basingstoke, UK). All other chemicals were obtained from Tianjin Jiangtian Chemical Technology Co., Ltd. (Tianjin, China).

### 2.2. Cultivation

Fifty microliters of *Schizochytrium* ATCC 20888 culture were inoculated into solid plates and cultivated at 30°C until colonies appeared. Then, a single colony was inoculated into 20 mL of seed mediums and cultivated at 28°C for 48 h to recover the growth of *Schizochytrium* ATCC 20888. At last, ~800 μL of seeds cultures were inoculated into 100 mL shaking flasks containing 20 mL of fermentation medium with an initial OD_630_ at 0.3 measured on ELX808 microplate reader (BioTek Instruments, America) for 60 h. The composition of liquid seed medium is: glucose 5.0 g/L, yeast extract 1.0 g/L, peptone 1.0 g/L, sea salt 20.0 g/L, pH 6.5. The fermentation medium is consisted of glucose 40.0 g/L, Na_2_SO_4_ 10.0 g/L, (NH_4_)_2_SO_4_ 0.8 g/L, KH_2_PO_4_ 4.0 g/L, KCl 0.2 g/L, MgSO_4_ 7H_2_O 4.1 g/L, sodium glutamate 20.0 g/L, CaCl_2_•2H_2_O 0.1 g/L, yeast extract 0.8 g/L, pH 5.5. 15.0 g/L of agar is added for solid medium.

### 2.3. Treatment with chemical modulators

Propanol or 1,3-propanediol (1,3-PD) used in this study was sterilized by filtering through a 0.22 μm membrane. They were then added individually or combinedly into fermentation medium before cultivation. For the supplementation of single additive, the final concentration of propanol in medium was 2.0, 4.0, 6.0, 8.0, or 10.0 g/L, and the final concentration of 1,3-PD in medium was 10.0, 20.0, 30.0, 40.0, or 50.0 g/L, respectively. For the supplementation with mixed additives, the concentration ratio of propanol and 1,3-PD was 2.0–10.0, 2.0–20.0, 2.0–30.0, 2.0–40.0, 4.0–10.0, 4.0–20.0, 4.0–30.0, 4.0–40.0, 6.0–10.0, 6.0–20.0, 6.0–30.0, or 6.0–40.0 g/L, respectively.

### 2.4. Biomass, glucose, fatty acid and reactive oxidative species measurements

Five hundred microliters of cultures at 24, 48, or 60 h were added into a pre-weighed 1.5 ml tube and the supernatants were removed by centrifugating at 20,817× *g* for 10 min. The residues were washed with H_2_O, and frozen in a −80°C refrigerator for 12 h and then placed in a vacuum freeze dryer (Beijing Songyuan Huaxing Technology Development Co., Ltd., China) for 12 h. The dry cell weight was determined by the gravimetric method.

The supernatant of culture was transferred into a clean 1.5 mL of tube to measure residual glucose content based on the glucose oxidase method. The working steps followed the protocols of the Glucose Oxidase Assay Kit (Biosino Biotechnology and science Co., Ltd., China).

Fatty acids methyl esters (FAMEs) were prepared according to the following methods. The cultures were collected at the 60 h and centrifuged at 7,197× *g* for 10 min. The supernatant was discarded, and the sediment was placed in a −80°C refrigerator for 12 h. The sediment dried in a vacuum freeze dryer (Beijing Songyuan Huaxing Technology Development Co., Ltd., China) for 12 h was used for methyl esters treatment. Approximately 20.0 mg of microalgal dry powder was weighted and dissolved in 2.0 mL of chloroform and 2.0 mL of esterification solution (methanol containing 3.0% (*v*/*v*) sulfuric acid and 0.5 mg/mL methyl nonadecanoate as an internal standard). The reaction was carried out at 97°C for 120 min and added 3.0 mL of distilled water to separate the water phase from chloroform phase. The chloroform phase was used for fatty acid measurement by a GC–MS system-GC 7890 coupled to an MSD 5975 (Agilent Technologies, Inc., Santa Clara, CA) equipped with a HP-5MS capillary column (30 m × 250 mm id). The FAMEs were identified by using NIST 11 mass spectral library (NIST/EPA/NIH mass spectral library, 2011 edition) and the fatty acid content was determined using standard curve methods with methyl nonadecanoate as an internal standard.

The reactive oxidative species (ROS) level was quantified by ROS Fluorescence Test Kit (Elabscience Biotechnology Co., Ltd., Shanghai, China). Briefly, equal amounts of *Schizochytrium* cells were resuspended in phosphate buffer containing 1 μL of DCFH-DH probe. The mixture was incubated at 37°C for 30 min and then washed with phosphate buffer for three times. The ROS content was quantified by a fluorescence spectrometer (excitation: 488 nm; emission: 525 nm).

### 2.5. GC–MS based metabolomics and WGCNA analysis

Approximately 10.0 OD_660_ of cells at 60 h were collected and immediately frozen in liquid nitrogen. The extraction and derivatization methods followed the methods described else (Lv et al., 2020). The GC–MS analysis was performed on a GC–MS system-GC 7890 coupled to an MSD 5975 (Agilent Technologies, Inc., Santa Clara, CA) equipped with an HP-5MS capillary column (30 m × 250 mm id). Data processing and statistical analysis was carried out using the Automated Mass Spectral Deconvolution and Identification System. The metabolomic data was normalized by the internal control and the cell numbers of the samples. The SIMCA-P 11.5 software was used for principal component analysis (PCA).

WGCNA was carried out for constructing a metabolic correlation network. First, calculating the weighted Pearson correlation matrices corresponding to metabolite abundance. Then creating the network using the methods described else (Zhang and Horvath, 2005). The highly similar correlated metabolites were grouped into modules by hierarchical clustering based on topological overlap. At last, the modules with correlation coefficient *r* > 0.6 and value of *p* < 0.05 were chosen for further analysis (Su et al., 2014; Li et al., 2017).

### 2.6. Triacylglycerol analysis

*Schizochytrium* cells were collected at 60 h and freeze-dried into a lyophilized algal powder. Total lipid content was extracted from 20 mg of lyophilized algal powder using a chloroform: methanol (*v*/*v*, 2:1) solution. The process was repeated for two times, and the extracts was washed with 2.0 mL of 1.0 M KCl and double-distilled water. The solvents were removed using a vacuum concentrator system (ZLS-1, Hunan, China). The dried extracts were dissolved in chloroform to a final concentration of 20.0 mg/ml and spotted on a Silica Gel 60 TLC plates (20 cm × 20 cm). The dried lipid spots were separated with a solvent system containing n-hexane: absolute ether: acetic acid (*v*/*v*/*v*, 70:30:1), the developed TLC plates were air dried and sprayed with 8% (*w*/*v*) H3PO4 containing 10% (*w*/*v*) copper (II) sulfate pentahydrate and charred at 180°C for 10 min (Yoon et al., 2012). The bands corresponding to triacylglycerol were identified by standard samples and collected by scraping the silica into a glass tube. The fatty acid analysis of triacylglycerol was prepared followed the methods described above.

### 2.7. Statistical analysis

All the experiments were carried out with at least three biological replicates. A statistical *T*-test was applied for statistical analysis and value of *p* less than 0.05 was considered as a threshold of significance.

## 3. Results and discussion

### 3.1. Effect of propanol supplementation on growth and fatty acid production in *Schizochytrium* ATCC 20888

**Figure 1:**
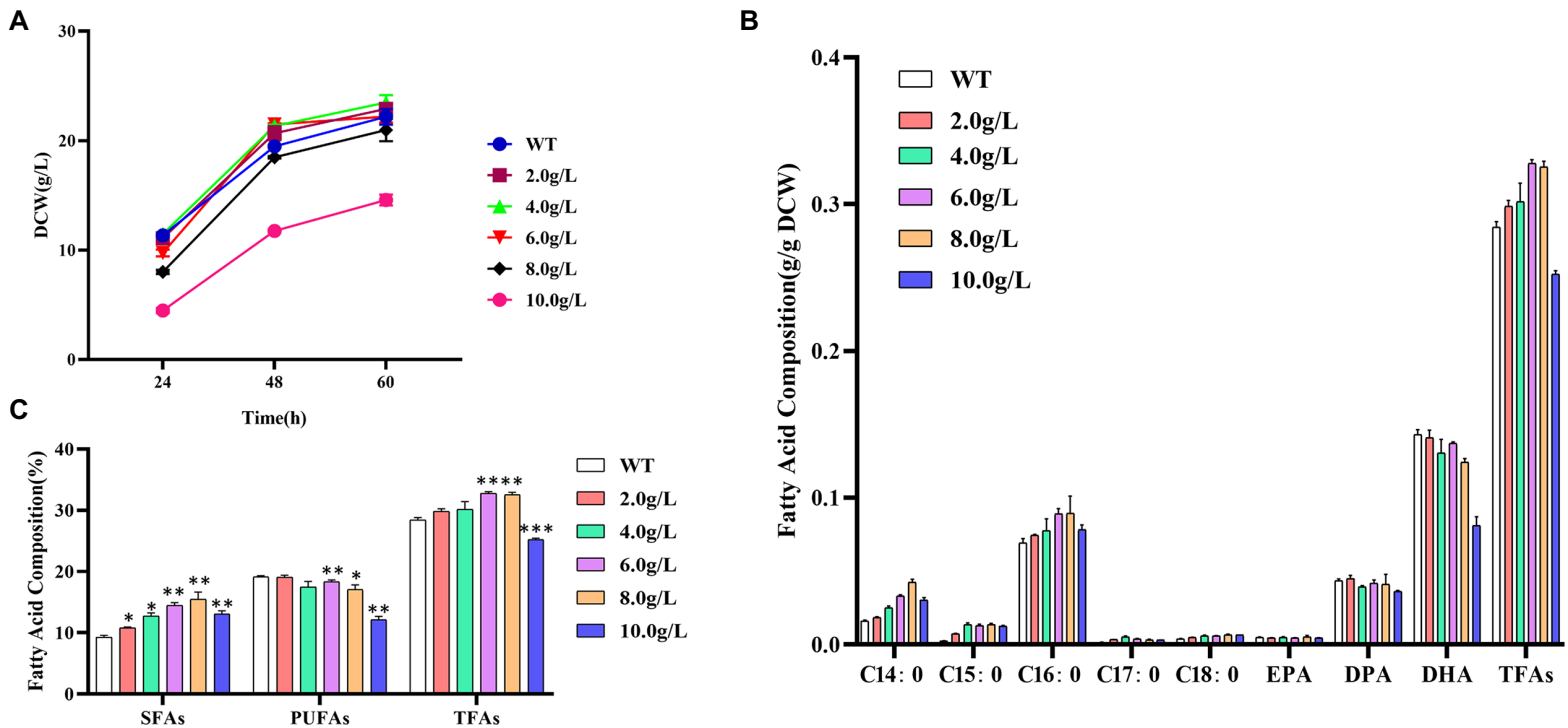
Effects of different concentrations of propanol on *Schizochytrium* ATCC 20888. **(A)** Growth curves; **(B)** Fatty acid composition; **(C)** Composition of saturated fatty acids (SFAs), polyunsaturated fatty acids (PUFAs) and total fatty acids (TFAs). *p-value <0.1, **p-value <0.05, ***p-value <0.01.

As shown in Figure 1A, the addition of 2.0, 4.0, or 6.0 g/L of propanol issued no significant effect on the biomass accumulation, but when it reached 8.0 g/L, the growth inhibition gradually occurred. The dry cell weight of 10.0 g/L addition at 60 h was only 65.7%, comparing with the control without propanol.

The content of C14:0, C15:0, C16:0, C17:0, C18:0 or EPA in dry cell weight was increased, while DPA or DHA was decreased with the elevation of propanol titer from 2.0 to 8.0 g/L in *Schizochytrium* ATCC 20888, respectively (Figure 1B). The content of total saturated fatty acids in dry cell weight was significantly elevated after addition of 2.0 g/L of propanol and the polyunsaturated fatty acid (EPA, DPA, and DHA) content in dry cell weight was not decreased until 6.0 g/L of propanol (Figure 1C). Since the increased extent of total saturated fatty acids was larger than the decreased extent of polyunsaturated fatty acid, the content of total fatty acid was improved from 2.0 to 6 g/L of propanol. Compared with the control, the total fatty acids and saturated fatty acid at 6.0 g/L of propanol achieved the highest elevation, elevating 15.3 and 55.4%, respectively. Together, the results suggested that propanol, at the concentration from 2.0 to 8.0 g/L, issued negligible effect on cell growth of *Schizochytrium* ATCC 20888, but exerted a positive effect on the saturated fatty acids accumulation, and negative effect for polyunsaturated fatty acids synthesis.

Methanol at low concentration (1.6–3.2%, *v/v*) has little effect on fatty acids biosynthesis, but it inhibited fatty acid biosynthesis significantly at high concentration (>4.8%, *v*/*v*) in *S*. *limacinium* B4D1 (Du et al., 2019). The addition of ethanol (1.0–3.0%, *v*/*v*) increased saturated fatty acids but affected little on polyunsaturated fatty acids in *S*. *limacinium* B4D1 (Zhu et al., 2022). *S*. *limacinium* B4D1 synthesized increased levels of saturated fatty acids accompanied by decreased polyunsaturated fatty acid levels at the addition of 2.0–8.0 g/L of butanol (Zhang et al., 2017). However, methanol, ethanol and butanol inhibited growth of *S*. *limacinium* B4D1 at all the tested concentrations. Compared with these reports, the advantage of addition of propanol is obvious as it stimulated total fatty acid accumulation without sacrificing biomass accumulation at the low concentration.

Zhang et al. (2019) reported that propanol could converted into propionyl-CoA to promote the synthesis of odd-chain saturated fatty acids (OCFAs) but decreased the even-chain fatty acid content in *Rhodococcus opacus* PD6300. In this study, we found that the addition of propanol increased OCFAs content by 2.8–4.9-fold, and C14:0 and C16:0 contents by 1.1–2.6-fold or 1.1–1.3-fold, respectively. Therefore, the mechanism of propanol to elevate fatty acids content in *Schizochytrium* was likely to be different from that in *R*. *opacus* PD630.

### 3.2. Effect of 1,3-PD addition on growth and fatty acid biosynthesis in *Schizochytrium* ATCC 20888

**Figure 2:**
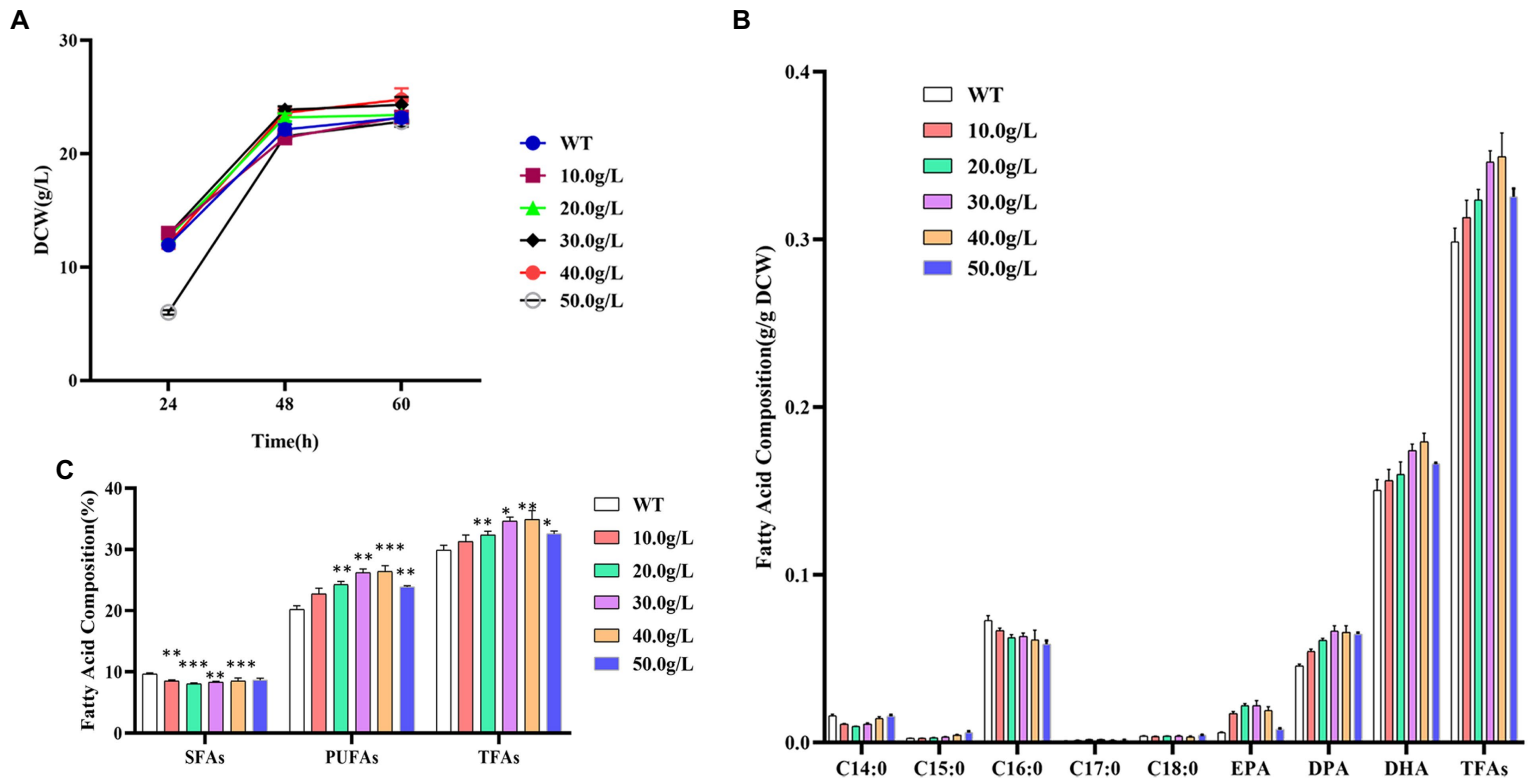
Effects of different concentrations of 1,3-propanediol on *Schizochytrium* ATCC 20888. **(A)** Growth curves; **(B)** Fatty acid composition; **(C)** Composition of saturated fatty acids (SFAs), polyunsaturated fatty acids (PUFAs) and total fatty acids (TFAs). *p-value <0.1, **p-value <0.05, ***p-value <0.01.

The dry cell weight of *Schizochytrium* ATCC 20888 was slightly increased when supplementing 20.0, 30.0, or 40.0 g/L of 1,3-PD (Figure 2A). The growth of *Schizochytrium* ATCC 20888 was obviously lagged after addition of 50.0 g/L of 1,3-PD at 24 h, but it achieved roughly the same biomass accumulation as the control, at the end of the fermentation. The observed change could not be due to the change of fermentation volume, because the maximum addition volume was only 800 μL, only accounting for 4.0% of the total fermentation volume.

The contents of the main saturated fatty acids C14:0 and C16:0 in dry cell weight were significantly decreased when adding 1,3-PD into the medium. For polyunsaturated fatty acid, the contents of EPA, DPA or DHA in dry cell weight was elevated after supplementing 1,3-PD into medium. In addition, the effect was dose-dependent, and the highest content was achieved when the concentration of 1,3-PD was over exceeded 30.0 g/L. Among them, EPA has the largest increase, which was 3.7 times higher than that of the control, reaching 515.2 mg/L. The EPA, DPA or DHA contents in dry cell weight was obviously decreased at 50.0 g/L of 1,3-PD. The increased extent of polyunsaturated fatty acids was larger than the decreased extent of saturated fatty acids, and total fatty acid content in dry cell weight was also promoted after 1,3-PD addition (Figure 2B). The effect of 1,3-PD on polyunsaturated fatty acids and total fatty acid was also dose-dependent. *Schizochytrium* ATCC 20888 achieved the highest polyunsaturated fatty acids and total fatty acid accumulation at 40.0 g/L of 1,3-PD addition, which was 30.7 and 17.0% higher than that without supplementation. Furthermore, due to the slight increase of dry weight, the titer of polyunsaturated fatty acids or total fatty acid was increased, 39.7% or 24.9% higher than that without supplementation, reaching 6.5 or 8.6 g/L at 40.0 g/L of 1,3-PD (Figure 2C). Together, these results suggested that the 1,3-PD, at the concentration range from 10.0 to 40.0 g/L, issues no effect on the growth of *Schizochytrium* ATCC 20888, and has positive and dose-dependent effect on polyunsaturated fatty acids and total fatty acid accumulation, but exerts adverse effect on C14:0 and C16:0 synthesis.

1, 3-propanediol, a glycerol derivative, is a good natural alternative to propylene glycol (Bertrand et al., 2018; Majumder et al., 2022). As annual market for 1,3-propanediol is over 100 million pounds and grows increasingly, most studies focused on production from glycerol or glucose by bacteria or yeast (Yang et al., 2018). Due to its chemical characteristics, the increase of 1, 3-propanediol in cells might be related to regulate redox balance and osmoregulation (Wang et al., 2015). To our knowledge, our study is the first report on exploring the potential of 1, 3-propanediol as a chemical modulator for stimulating high-valued fatty acid production in microalgae.

### 3.3. Exploring possible mechanisms for improving fatty acid content after propanol or 1,3-PD addition

**Figure 3:**
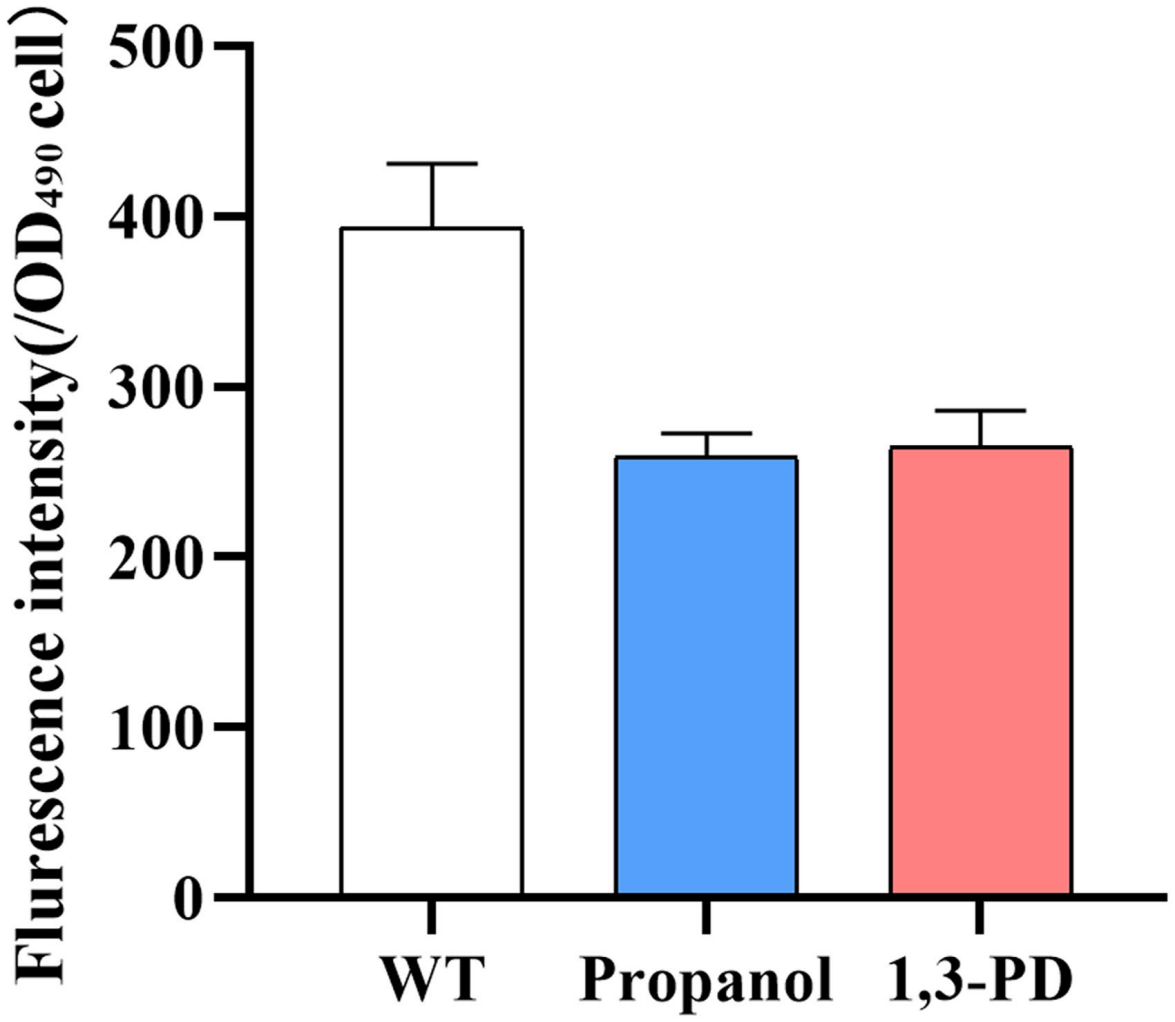
Analysis of ROS level under 6.0 g/L of propanol or 40.0 g/L of 1,3-PD.

The 1,3-propanediol has been reported to function in the regulation of redox balance and osmotic regulation in *Candida tropicalis* (Wang et al., 2015). Disturbing redox balance led to production of ROS, which could be detrimental to biomass and lipid accumulation in *Schizochytrium* (Schonfeld and Wojtczak, 2008). Decreasing ROS could decrease peroxidation of lipids and resulted in improving lipid yield in *Schizochytrium* (Sun et al., 2018a,b). Several strategies have been developed for alleviate ROS for increasing lipid content. For example, addition of antioxidants, such as ascorbic acid or mannitol for enhancing fatty acid production (Ren et al., 2017; Han et al., 2021); overexpression of oxidative stress defense genes to promote cell growth and lipid yield (Zhang et al., 2018, 2021). In our study, the ROS level was determined in *Schizochytrium* after adding 6.0 g/L of propanol or 40.0 g/L of 1,3-PD. It was found ROS levels was significantly decreased under both conditions (Figure 3). Therefore, the results suggested that the decrease of ROS levels could be one of possible factors enhancing fatty acid accumulation by propanol or 1,3-PD.

The concentration of propanol and 1,3-PD were also determined during cultivation. It was discovered that 1,3-propanediol was hardly used by *Schizochytrium*, while the concentration of propanol was decreased from 6.0 to 4.0 g/L after 48 h cultivation, suggesting propanol, not1,3-propanediol, can be used as substrates for *Schizochytrium*.

GC–MS-based metabolomics was applied to further explore possible mechanisms since the fatty acid profiles of 6.0 g/L of propanol and 40.0 g/L of 1,3-PD were different. Totally, 48 metabolites were confidentially identified, and chemically classified as amino acids, sugars, organic acids, and so on (see Supplementary material). The principal component analysis (PCA) showed that three biological replicates of each sample were clustered, while the samples were well separated. In addition, the metabolomic profile of 1,3-PD tended to be far away from that of WT and propanol, which suggested that significant metabolic changes occurred after 1,3-PD treatment.

**Figure 4:**
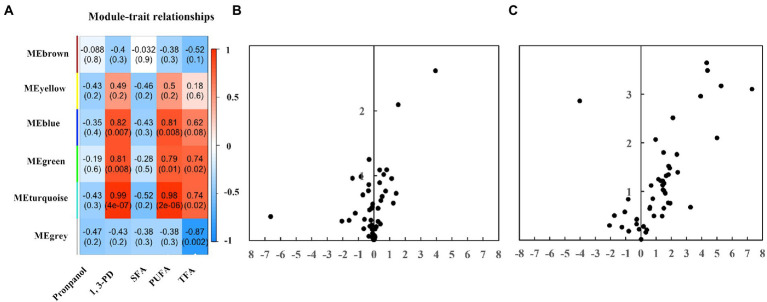
The GC–MS metabolomic profiling. **(A)** The heatmaps. The numbers within the heat map represent correlations and *p*-values for the module-trait associations. **(B)** A volcano plot analysis of the metabolic profile of propanol. **(C)** A volcano plot analysis of the metabolic profile of 1,3-PD.

A WGCNA network analysis was performed on GC–MS metabolomics datasets to identify modules responsive to chemicals modulators and fatty acids (Figure 4A). No module is associated with propanol and saturated fatty acids, suggesting less significant changes at the metabolome level after propanol addition. Module blue identified was found positively related to 1,3-PD and polyunsaturated fatty acids, including two alcohols (i.e., 1,2-ethanediol, inositol), two carboxylic acids (i.e., gluconic acid, acetic acid), three fatty acids (i.e., hydroxy-2,3-didehydrosebacic acid, oleic acid, octadecadiynoic acid), one amino acid (i.e., glutamic acid). Module green discovered was found to be positively associated with 1,3-PD, polyunsaturated fatty acids and total fatty acids, including two amino acids (i.e., serine, aspartic acid) and one carboxylic acid (i.e., phosphoric acid). Module turquoise was found positively associated with 1,3-PD, polyunsaturated fatty acids and total fatty acids, including two carboxylic acids (i.e., propanoic acid, galacturonic acid), one ane (i.e., decane), five amino acids (i.e., glycine, leucine, threonine, citrulline, glutamine), two alcohols (i.e., glycerol, 1,2-propanediol-1-phosphate), seven sugars (i.e., ribopyranose, tagatose, glucopyranose, talofuranose, allose, glucopyranoside, galactose), three fatty acids (i.e., octadecanoic acid, monopalmitoylglycerol, tetradecanoic acid), and one cyclopentene. The differentially expressed metabolites were also exhibited by a volcano plot (Figure 4C). Setting fold change greater or lower than 2 and *p* value was less than 0.05, two compounds, tagatose and tetradecanoic acid, were significantly upregulated under 6.0 g/L of propanol; fourteen compounds were significantly elevated while ribopyranose was significantly downregulated under 40.0 g/L of 1,3-PD. Together, these results suggested that 1,3-PD treatment could lead to significant changes at metabolome level in *Schizochytrium* ATCC 20888.

The metabolic differences caused by 1,3-PD were in consistent with the physiological alternation of *Schizochytrium* ATCC 20888: (i) Osmoregulation: glycerol prevents osmotic water loss and mitigates environmental stress such as freezing and hyperomostic conditions in microalgae like *Dunaliella*, *Chalmydomonas* or *Brachiomonas submarina* (Ahmad and Hellebust, 1985; Raymond et al., 2020). Glutamic acid and glutamine are also osmoregulators after hyperosmotic shock in *Brevibacterium lactofermentum*, *Corynebacterium glutamicum* and *Escherichia coli* (Larsen et al., 1987; Skjerdal et al., 1996). All of these metabolites were found positively associated with 1,3-PD treatment. In addition, glycerol and glutamine were significantly elevated by 3.2 and 2.8-fold at 40.0 g/L of 1,3-PD, suggesting that *Schizochytrium* ATCC 20888 was suffered from possible hyperosmotic stress; (ii) Triacylglycerol biosynthetic pathway: two major pathways, glycerol phosphate pathway and monoacylglycerol pathway, have been proposed to function in *Schizochytrium* (Zienkiewicz et al., 2016; Limei et al., 2022). Glycerol and monopalmitoylglycerol, the precursors of glycerol phosphate pathway and monoacylglycerol pathway, were found positively associated with polyunsaturated fatty acids and total fatty acids. In addition, they were significantly elevated after 1,3-PD addition, suggesting that triglyceride biosynthetic pathway was likely to be activated; (iii) Amino acids: WGCNA analysis showed that aspartic acid, glutamine and leucine were positively responsive to 1,3-PD treatment. The dry cell weight of *Schizochytrium* was slightly increased in 1,3-PD condition. It was reported that better biomass accumulation can be achieved for *Schizochytrium* growth on medium containing aspartic acid, glutamine and leucine (Patil and Gogate, 2015). Therefore, it is speculative that upregulation of these amino acids is related to biomass accumulation.

**Figure 5:**
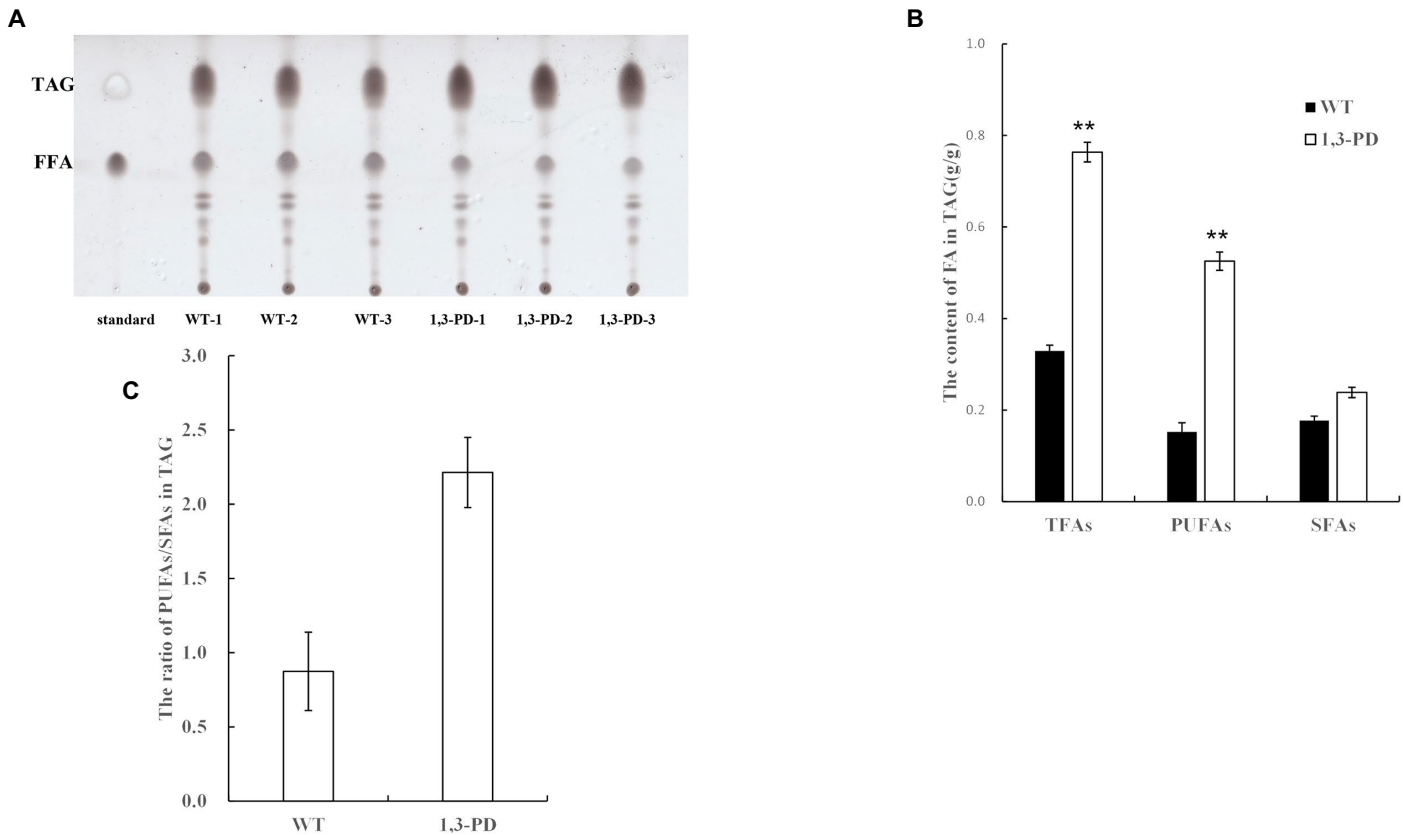
Comparison of the content and fatty acid profiles of triacylglycerol (TAG) with/without supplementing 1,3-PD.**(A)** The separation result of lipid by TLC. **(B)** The contents of FA in triacylglycerol. **(C)** The ratio of PUFAs/SFAs in triacylglycerol. *p-value <0.1 **p-value <0.05 ***p-value <0.01.

It was reported that diacylglycerol acyltransferases, which catalyze the final and committed step of triacylglycerol, have specificity toward acyl-CoA, further determining fatty acid profile of triacylglycerol in microalgae (Xin et al., 2019; Ma et al., 2021). As triacylglycerol is the dominant form of fatty acids in microalgae, we proposed a plausible hypothesis that the content of glycerol was elevated in *Schizochytrium* to resist hyperosmotic stress provoked by 1,3-PD, resulting in the activation of triacylglycerol biosynthesis pathway. Enzymes in triacylglycerol biosynthesis pathway is possible to have PUFA-CoA substrate preference, forcing enhancing PUFA synthesis in *Schizochytrium*. GC–MS-based metabolomics has also demonstrated that *Schizochytrium* migh be suffered from hyperosmotic stress, and then the content and profile of triacylglycerol was quantified in *Schizochytrium* with/without adding 1,3-PD. As shown in Figure 5A, the triacylglycerol content was significantly increased after supplementing 40.0 g/L of 1,3-PD. These results suggested that triacylglycerol pathway might indeed be activated. The TAG-associated fatty acid profiles were exhibited in Figure 5B. It was discovered that the ratio of PUFAs to SFAs was much higher when 1,3-PD was added, also suggesting the preference of triacylglycerol pathway after supplementing 1,3-PD. Together, these evidences partly supported the hypothesis we proposed. However, further work is still needed to determine the exact mechanism, including upregulated genes responsible for acyl-CoA preference.

### 3.4. Cumulative effects of propanol and 1,3-PD on growth and fatty acid accumulation

It is well known that a combined application of different chemical modulators may exert synergistic effect on fatty acid accumulation, especially when their mechanisms of regulating fatty acids are different (Wang et al., 2018). As mentioned above, we speculated that propanol and 1,3-PD may function on enhancing fatty acid accumulation by different mechanisms. Therefore, propanol and 1,3-PD were combined to further explore the effects on cell growth and fatty acid accumulation in *Schizochytrium* ATCC 20888. As the concentration of propanol or 1,3-PD that not compromising growth was from 2.0 to 6.0 g/L or 10.0 to 40.0 g/L, varying concentration of propanol within the range was combined with different concentration of 1,3-PD within the range to evaluate the effects on cell growth and fatty acid accumulation of *Schizochytrium* ATCC 20888.

**Figure 6:**
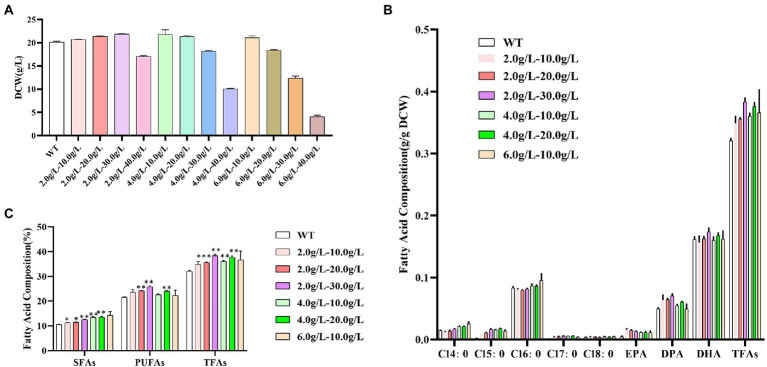
Effects of different concentrations of mixed propanol and 1,3-PD on *Schizochytrium* ATCC 20888. **(A)** Dry cell weight at 60 h; **(B)** Fatty acid composition; **(C)** Composition of saturated fatty acids (SFAs), polyunsaturated fatty acids (PUFAs) and total fatty acids (TFAs). *p-value <0.1, **p-value <0.05, ***p-value <0.01.

As shown in Figure 6A, at the concentration of 2.0 g/L of propanol, the dry cell weight of *Schizochytrium* ATCC 20888 was slightly increased when the concentration of 1,3-PD was from 10.0 to 30.0 g/L and sharply decreased at the concentration of 40.0 g/L. At the concentration of 4.0 g/L of propanol, the biomass was not decreased until the concentration of 1,3-PD reached 30.0 g/L. At the concentration of 6.0 g/L of propanol, the growth of *Schizochytrium* ATCC 20888 was not inhibited until 10.0 g/L of 1,3-PD. Therefore, the combination of propanol and 1,3-PD at 2.0–10.0, 2.0–20.0, 2.0–30.0, 4.0–10.0, 4.0–20.0, and 6.0–10.0 g/L were further chosen to determine the effects on fatty acid accumulation.

As shown in Figures 6B,C, the contents of C14:0, C16:0 and the total saturated fatty acids were increased with the elevation of propanol titer in propanol &1,3-PD combination. There seemed little promotion of C14:0, C16:0 or total saturated fatty acids content when improving 1,3-PD titer in propanol &1,3-PD combination. The highest contents of C14:0, C16:0 and the total saturated fatty acids were achieved at 6.0 g/L of propanol with 10.0 g/L of 1,3-PD, which was 72.8, 14.6, and 14.1% higher than the control, respectively. But it was still lower than that with 6.0 g/L of propanol alone. The content of DPA, DHA and polyunsaturated fatty acids was increased at 2.0–20.0, 2.0–30.0, or 4.0–20.0 g/L of propanol and 1,3-PD combination. There seemed a 1,3-PD dose-dependent effect at 2.0 or 4.0 g/L of propanol in propanol &1,3-PD combination. The highest DPA, DHA and polyunsaturated fatty acid contents were obtained at 2.0 g/L of propanol & 30.0 g/L of 1,3-PD, which were still lower than supplementation of 40.0 g/L of 1,3-PD. The total fatty acids content was significantly promoted in all propanol &1,3-PD combination. In consistent with polyunsaturated fatty acid, the total fatty acids content was increased with elevation of 1,3-PD concentration at 2.0 or 4.0 g/L of propanol in propanol &1,3-PD combination. The combination of 2.0 g/L of propanol and 30.0 g/L of 1,3-PD achieved the maximum increase of total fatty acids, reaching 1.2-fold, which was greater than that of the two substances added alone. In addition, no growth decrease was observed for any of the combined supplementing experiments for *Schizochytrium*. Together, our results suggested that there exist synergetic effects of propanol and 1,3-PD on stimulating total fatty acids accumulation. Compared with previous reports in *Schizochytrium* (Supplementary Table S2), the fatty acid titer achieved in this study was relatively higher. Furthermore, this study could also be supplied designer lipid with tailored application purposes. The methods we described not only increased total fatty acids contents, but also supplied. In addition, it is a scalable process since chemical modulators is easily applied in large-scale production.

## 4. Conclusion

In this study, fatty acid was significantly increased after addition of chemical modulator propanol or 1,3-PD into the *Schizochytrium* ATCC 20888 culture. Propanol increased saturated fatty acids content, while 1,3-PD promoted polyunsaturated fatty acids accumulation. GC–MS based metabolomics showed that the mechanisms of elevating fatty acids are possibly different between propanol and 1,3-PD, despite both of them could quench intracellular ROS. Propanol did not lead to obviously metabolic change while 1,3-PD elevated contents of osmoregulators and amino acids and activated triacylglycerol pathway. Biochemical evidences showed that the content and fatty acid profile of triacylglycerol were significantly changed, which explains the preference on fatty acid profile in *Schizochytrium* ATCC 20888 after addition of 1,3-PD. In addition, the two chemical modulators have synergistic effects, since the total fatty acids can be improved to the greatest extent, elevated 1.2-fold by simultaneously addition of propanol and 1,3-PD. By adjusting the proportion of the two, the ratio of saturated fatty acids and polyunsaturated fatty acids in total fatty acids can be changed. As propanol and 1,3-PD are both easily applied in large-scale production, the whole process is expected to scale up to an industrial level in further. These findings provide new strategy for producing tailored fatty acids profile for satisfying needs in *Schizochytrium* using chemical modulators.

## Data availability statement

The original contributions presented in the study are included in the article/Supplementary material, further inquiries can be directed to the corresponding authors.

## Author contributions

TW conceptualization, investigation, methodology, formal analysis, and data curation. FW conceptualization, investigation, methodology, formal analysis, data curation, funding, supervision, writing—original draft, and writing—review and editing. LZ methodology and data curation. PG data curation, writing—review and editing. YW writing —review and editing. LC investigation, formal analysis, supervision, and writing—review and editing. WZ conceptualization, formal analysis, funding, supervision, and writing—review and editing. All authors contributed to the article and approved the submitted version.

## Funding

This research was supported by grants from the National Key R&D Program of China (Nos. 2020YFA0908703 and 2019YFA0904600) and the National Natural Science Foundation of China (No. 32101172).

## Conflict of interest

The authors declare that the research was conducted in the absence of any commercial or financial relationships that could be construed as a potential conflict of interest.

## Publisher’s note

All claims expressed in this article are solely those of the authors and do not necessarily represent those of their affiliated organizations, or those of the publisher, the editors and the reviewers. Any product that may be evaluated in this article, or claim that may be made by its manufacturer, is not guaranteed or endorsed by the publisher.
