# Supplementary material for: Propanol and 1, 3-propanediol enhance fatty acid accumulation synergistically in Schizochytrium ATCC 20888
Source: Front Microbiol. 2023 Feb 9;13:1106265. doi: 10.3389/fmicb.2022.1106265 (PMC9947470; doi:10.3389/fmicb.2022.1106265)
Supplement: Supplementary file 1 [file Data_Sheet_1.docx]

Supplementary Material

Propanol and 1, 3-propanediol enhance fatty acid accumulation synergistically in *Schizochytrium* ATCC 20888

**Tiantian Wang ^1,2^, Fangzhong Wang ^3*^, Lei Zeng ^1,2^, Pengfei Guo ^1,2^, Yawei Wu ^1,2^, Lei Chen ^1,2*^, Weiwen Zhang ^1,2,3^**

^1^Laboratory of Synthetic Microbiology, School of Chemical Engineering and Technology, Tianjin University, Tianjin, PR China

^2^Frontier Science Center of Synthetic Biology and Key Laboratory of Systems Bioengineering (Ministry of Education), Tianjin University, Tianjin, PR China

^3^Center for Biosafety Research and Strategy, Tianjin University, Tianjin, PR China

***Correspondence:**

Fangzhong Wang,
[fangzhong.wang@tju.edu.cn](mailto:fangzhong.wang@tju.edu.cn) (F. W.)

Lei Chen,

lchen@tju.edu.cn (L.C.)


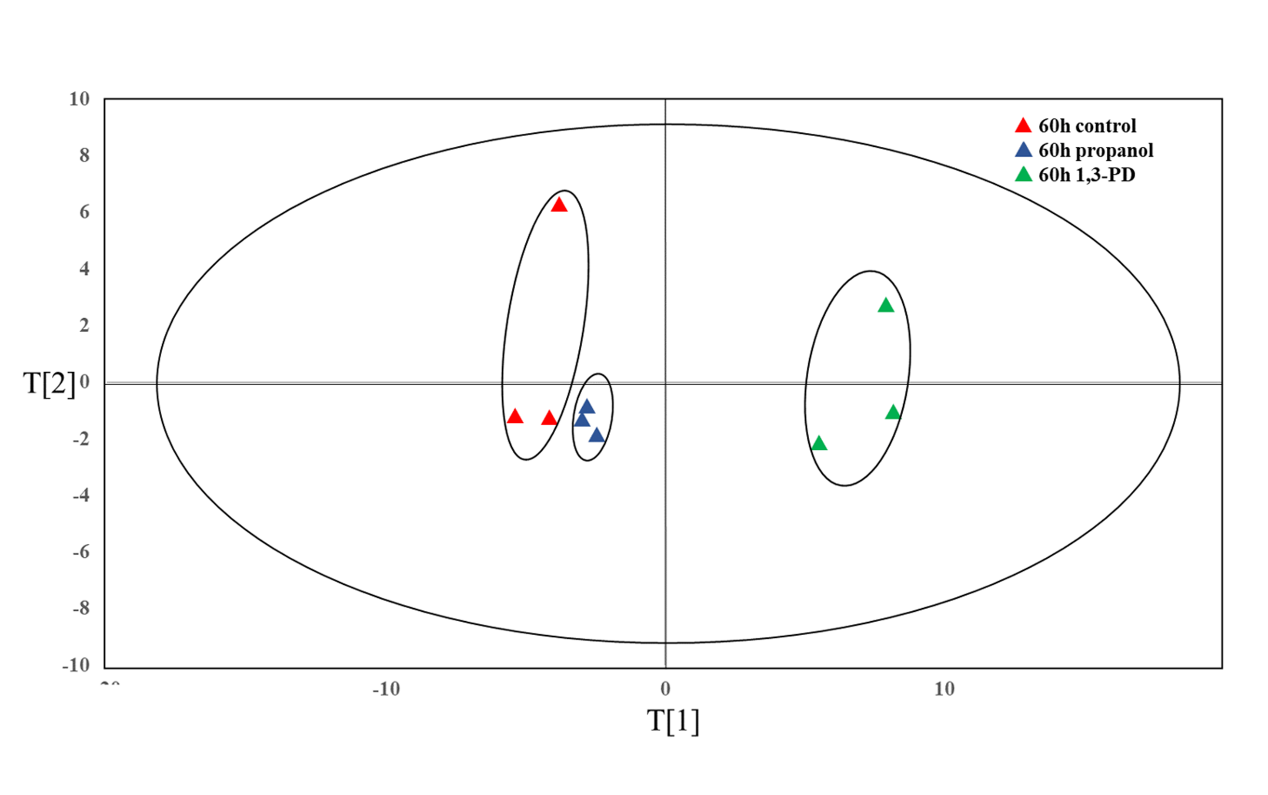


**Figure S1** **PCA analysis of GC–MS metabolomic data**. T1 and T2 were considered as score in principal component 1st and 2nd, respectively.

**Table S1** GC–MS metabolomic data set of *Schizochytrium* with/without addition of propanol or 1,3-PD.

|  | WT-60-1 | WT-60-2 | WT-60-3 | Propanol-60-1 | Propanol-60-2 | Propanol-60-3 | 1,3-PD-60-1 | 1,3-PD-60-2 | 1,3-PD-60-3 |
| --- | --- | --- | --- | --- | --- | --- | --- | --- | --- |
| Propanoic acid | 0.60335471 | 1.31493502 | 0.8815571 | 1.15755316 | 1.46065012 | 2.06526936 | 2.95513777 | 1.88453545 | 3.14423915 |
| Alanine | 1.0508686 | 1.5375057 | 0.97902806 | 1.30502258 | 1.20760498 | 1.17509011 | 1.02019753 | 0.86999164 | 1.05776626 |
| 1,2-ethanediol | 0.01590574 | 0.0172108 | 0.26039809 | 0.24381367 | 0.22834375 | 0.22073158 | 1.76987135 | 0.55652869 | 0.498702 |
| gluconic acid | 0.02125615 | 0.01914261 | 0.02262323 | 0.02054997 | 0.03196371 | 0.03828771 | 0.14977208 | 0.08421958 | 0.10410388 |
| decane | 0.04432113 | 0.03610033 | 0.06392689 | 0.04373235 | 0.05868652 | 0.06936127 | 0.21992451 | 0.18978603 | 0.21034322 |
| acetic acid | 0.59032589 | 0.47871364 | 0.59085834 | 0.52779603 | 0.48699034 | 0.5513671 | 2.7692539 | 0.990978 | 0.9564512 |
| glycine | 0.08026233 | 0.15165697 | 0.08631054 | 0.10244993 | 0.10201931 | 0.0921442 | 0.24241871 | 0.21720471 | 0.24737013 |
| leucine | 0.14783173 | 0.2263997 | 0.15584361 | 0.11470136 | 0.08439554 | 0.12353899 | 0.27410764 | 0.21395293 | 0.30673001 |
| proline | 1.46180213 | 3.41045483 | 1.57946035 | 1.87528155 | 1.92020313 | 1.58373516 | 2.12151674 | 1.81764682 | 4.152667 |
| isoleucine | 0.09503242 | 0.12807506 | 0.07565304 | 0.04945732 | 0.05868344 | 0.05912209 | 0.04854549 | 0.01531424 | 0.07939245 |
| valine | 0.11681813 | 0.15085283 | 0.10229599 | 0.12186908 | 0.10198228 | 0.10973634 | 0.09752051 | 0.09719872 | 0.11235086 |
| inositol | 0.09505321 | 0.08327598 | 0.1110304 | 0.07457307 | 0.12327159 | 0.12300735 | 0.35360111 | 0.19912782 | 0.2285017 |
| serine | 0.19932579 | 0.37572435 | 0.21586352 | 0.26566992 | 0.26955637 | 0.26634624 | 0.36000923 | 0.37096361 | 0.46787346 |
| glycerol | 0.34835222 | 0.43882699 | 0.34071227 | 0.3165108 | 0.40786433 | 0.38328593 | 0.98081187 | 1.62456003 | 0.97149496 |
| threonine | 0.30413549 | 0.38899393 | 0.2982087 | 0.29010825 | 0.29316198 | 0.29380153 | 0.53558562 | 0.47352039 | 0.57168651 |
| butanedioic acid | 1.21000111 | 1.76202097 | 1.26694568 | 1.1878618 | 1.10121356 | 1.18194783 | 1.14920563 | 1.08019596 | 1.27498195 |
| uracil | 0.0085344 | 0.06382294 | 0.00147332 | 0.02596759 | 0.00945653 | 0.02310376 | 0.01966867 | 0.01255089 | 0.01025095 |
| monomethylene-l-rhamnitol | 0.00538632 | 0.0471102 | 0.0382475 | 0.01760453 | 0.01947637 | 0.02217889 | 0.04232797 | 0.0291177 | 0.04696153 |
| aspartic acid | 0.00617995 | 0.03081512 | 0.0235425 | 0.04716379 | 0.04939926 | 0.03585913 | 0.05590261 | 0.07073871 | 0.09766965 |
| 2-Pyrrolidone-5-carboxylic acid | 2.96916545 | 4.45028631 | 3.26975501 | 3.63921059 | 3.16460983 | 3.05610386 | 3.48684415 | 3.81588483 | 4.81388934 |
| cyclopentene | 0.27688505 | 0.37462224 | 0.21204916 | 0.32470902 | 0.33302317 | 0.24932415 | 0.6098146 | 0.83069104 | 1.03866993 |
| asparagine | 0.05763858 | 0.17852526 | 0.09410073 | 0.12475177 | 0.14250156 | 0.078922 | 0.12052881 | 0.16605438 | 0.31439292 |
| 1,2-propanediol-1-phosphate | 0.00588848 | 0.03115424 | 0.02121313 | 0.02139152 | 0.01437212 | 0.01848901 | 0.36728045 | 0.39727737 | 0.40109211 |
| glutamic acid | 0.63049272 | 0.82935896 | 0.67980104 | 0.90403257 | 0.87188114 | 0.97808633 | 1.54833072 | 1.00309854 | 1.13175475 |
| citrulline | 0.01885636 | 0.04419067 | 0.03113659 | 0.02421217 | 0.0365831 | 0.02119241 | 0.10842667 | 0.06239197 | 0.11657634 |
| glutamine | 0.70908323 | 0.95571271 | 0.54112226 | 0.96440454 | 1.04099135 | 0.83914017 | 1.98210585 | 2.02506259 | 2.18709791 |
| phosphoric acid | 0.69018625 | 1.88509976 | 0.97403513 | 1.68340767 | 2.13861563 | 1.65188314 | 2.33868283 | 2.87606206 | 3.49246298 |
| ribopyranose | 0.70923771 | 0.72059711 | 0.69479515 | 0.51524613 | 0.56949889 | 0.61951788 | 0.02746741 | 0.02439292 | 0.07870162 |
| tetradecanoic acid | 0.02778048 | 0.03143413 | 0.03409692 | 0.09219533 | 0.09773315 | 0.08324073 | 0.18959935 | 0.1345484 | 0.15714985 |
| 2-ketoglutaric acid | 0.1019649 | 0.11364073 | 0.10965895 | 0.09686711 | 0.09580429 | 0.11015837 | 0.11270591 | 0.07777269 | 0.1088696 |
| tagatose | 0.0150351 | 0.01757017 | 0.0135067 | 0.21866175 | 0.2357286 | 0.25463455 | 0.23611394 | 0.22303021 | 0.2439072 |
| glucopyranose | 0.07162212 | 0.08206915 | 0.16823897 | 0.42377785 | 0.2198781 | 0.21413707 | 0.4058151 | 0.40752815 | 0.320067 |
| glucose | 0.11602497 | 0.05984556 | 0.12547086 | 0.23203549 | 0.1358224 | 0.16152548 | 0.19495511 | 0.16864489 | 0.22635669 |
| pentadecanoic acid | 0.03294146 | 0.03658593 | 0.02447505 | 0.02909168 | 0.0228197 | 0.02093159 | 0.03029831 | 0.01972203 | 0.04533843 |
| tyrosine | 0.01973219 | 0.22343342 | 0.01598102 | 0.03201726 | 0.02740911 | 0.02546834 | 0.02608753 | 0.03608661 | 0.0463432 |
| monopalmitoylglycerol | 0.65451856 | 0.72246222 | 0.89796156 | 0.59968493 | 1.18026286 | 1.30833743 | 2.14027802 | 2.4336026 | 3.50666371 |
| talofuranose | 0.00102369 | 0.00170179 | 1.002838 | 0.00368206 | 0.0026275 | 0.00381709 | 0.93760336 | 0.99018447 | 0.71032648 |
| allose | 0.00277205 | 0.00543886 | 0.01407364 | 0.00152252 | 0.00571341 | 0.00242618 | 1.20896063 | 1.10728506 | 1.20608746 |
| oleic acid | 0.03895371 | 0.02981681 | 0.03306773 | 0.03216686 | 0.03958746 | 0.03242606 | 0.13708872 | 0.07107949 | 0.07156539 |
| octadecanoic acid | 0.36875569 | 0.4876661 | 0.69179737 | 0.24951099 | 0.7980692 | 0.8000144 | 0.82006384 | 1.45477103 | 1.91696693 |
| galacturonic acid | 0.08108006 | 0.1423922 | 0.07322542 | 0.21564684 | 0.02952927 | 0.03969782 | 2.072684 | 2.02341046 | 2.05404436 |
| uridine | 0.02246739 | 0.20061202 | 0.08871077 | 0.0930142 | 0.02433995 | 0.08012009 | 0.03785141 | 0.02771538 | 0.02769628 |
| hydroxy-2,3-didehydrosebacic acid | 0.03264716 | 0.03263504 | 0.04115185 | 0.04018996 | 0.02926657 | 0.03413059 | 0.20194714 | 0.06214356 | 0.10593814 |
| Octadecadiynoic acid | 0.02545249 | 0.04492001 | 0.0051995 | 0.02623487 | 0.03454492 | 0.01285458 | 0.16029031 | 0.06433171 | 0.06234074 |
| 2-α-Mannobiose | 0.01457404 | 0.01270886 | 0.6465191 | 0.04967497 | 0.05560326 | 0.05585825 | 0.04644748 | 0.05860278 | 0.05564754 |
| squalene | 0.61890993 | 0.30531462 | 0.38390519 | 0.15985343 | 0.16682829 | 0.17387121 | 0.26999216 | 0.15800294 | 0.30745032 |
| glucopyranoside | 0.00699437 | 0.02028991 | 0.02414849 | 0.02665742 | 0.02499143 | 0.01651085 | 0.6857483 | 0.65858654 | 0.64653555 |
| galactose | 0.03998685 | 0.05468658 | 0.03855472 | 0.02768012 | 0.04083047 | 0.03693441 | 1.6359518 | 1.224816 | 1.39782838 |

**Table S2** Comparison of fatty acid prodcution by *Schizochytrium* in shaking-flask culture

| Strain | Treatment | Dry cell weight  (g/L) | TFA titer(g/L) | References |
| --- | --- | --- | --- | --- |
| *Schizochytrium limacinum* SR21 | Ammonium sulfate | 24.4 | 2.0 | (Yokochi et al., 1998) |
|  | Ammonium acetate | 23.8 | 5.2 |  |
|  | Ammonium nitrate | 21.8 | 4.0 |  |
|  | Sodium nitrate | 14.6 | 2.8 |  |
|  | Urea | 24.8 | 2.6 |  |
| *Schizochytrium sp*. HX-308 | Cane molasses | 25.54 | 5.21 | (Yin et al., 2019) |
| *Schizochytrium sp.* BCRC33482 | Sugarcane bagasse | 10.45 | 4.72 | (Nguyen et al., 2018) |
| *Aurantiochytrium sp.* YLH70 | Jerusalem artichoke | 32.71 | 19.72 | (Yu et al., 2016) |
| *Schizochytrium limacinum* SR21 | Sweet sorghum juice (50%) | 9.38 | 6.86 | (Liang et al., 2010) |
| *Schizochytrium limacinum* SR21 | Crude glycerol + corn steep solid | 11.78 | 6.99 | (Ethier et al., 2011) |
| *Aurantiochytrium* sp. KRS101 | Empty palm fruit bunches | 34.40 | 12.50 | (Hong et al., 2013) |
| *Schizochytrium* sp. CGMCC8091 | Enteromorpha hydrolysate | 4.39 | 0.66 | (Yaodong Ning, 2020) |
| *Schizochytrium* PKU#Mn4 | Control | 5.89 | 1.57 | (Zhang et al., 2021) |
|  | Mannitol(1g/L) | 5.51 | 2.12 |  |
|  | Mannitol(1g/L)+Ascorbic acid(1g/L) | 8.49 | 3.79 |  |
|  | Mannitol(1g/L)+Ascorbic acid(2g/L) | 7.96 | 3.51 |  |
|  | Mannitol(1 g/L)+Ascorbic acid (3g/L) | 6.79 | 2.95 |  |
|  | Mannitol(1g/L)+Ascorbic acid(6g/L) | 4.67 | 2.08 |  |
|  | Mannitol(1g/L)+Ascorbic acid(9g/L) | 3.38 | 1.40 |  |
|  | Mannitol(1g/L)+Ascorbic acid(12 g/L) | 1.84 | 0.80 |  |
|  | Mannitol(1g/L)+Ascorbic acid (15g/L) | 1.22 | 0.58 |  |
| *Schizochytrium* sp. PKU#Mn4 | KH_2_PO_4_ (0.25g/L) | 7.15 | 2.86 | (Wang et al., 2018) |
|  | Salinity (40% seawater) | 9.54 | 2.24 |  |
|  | Temperature (28°C) | 9.12 | 1.63 |  |
|  | Initial pH 6.47 | 9.24 | 1.27 |  |
|  | Agitation speed (150rpm) | 8.76 | 1.10 |  |
|  | Glucose + Ethanol、YE | 13.72 | 5.28 |  |
| *Thraustochytriidae* sp. PKU#Mn16 | KH_2_PO_4_ (0g/L) | 9.17 | 1.72 |  |
|  | Salinity (40% seawater) | 9.08 | 2.59 |  |
|  | Temperature (28°C) | 8.43 | 2.92 |  |
|  | Initial pH 5.0 | 7.80 | 0.90 |  |
|  | Agitation speed (150rpm) | 9.42 | 1.04 |  |
| *Thraustochytrium* sp.AH-2 | Glucose (3%)+(NH_4_)_2_SO_4_ + Monosodium | 4.30 | 0.75 | (Thyagarajan et al., 2014) |
| *Aurantiochytrium* sp.TC20 | Glucose (2%)、Pep + YE | 12.0 | 4.84 | (Lee Chang et al., 2012) |
| *Schizochytrium* ATCC 20888 | Control | 22.17 | 6.53 | This study |
|  | Propanol (6.0g/L) | 22.20 | 7.71 | This study |
|  | 1,3-propanediol (40.0g/L) | 24.76 | 9.03 | This study |
|  | Propanol(2.0g/L)+1,3-propanediol (30.0g/L) | 21.9 | 8.47 | This study |

**References**

Ethier, S., Woisard, K., Vaughan, D., and Wen, Z. (2011). Continuous culture of the microalgae *Schizochytrium limacinum* on biodiesel-derived crude glycerol for producing docosahexaenoic acid. Bioresour. Technol. 102(1), 88-93. doi: 10.1016/j.biortech.2010.05.021.

Hong, W. K., Yu, A., Heo, S. Y., Oh, B. R., Kim, C. H., Sohn, J. H., et al. (2013). Production of lipids containing high levels of docosahexaenoic acid from empty palm fruit bunches by *Aurantiochytrium* sp. KRS101. Bioprocess. Biosyst. Eng. 36(7), 959-963. doi: 10.1007/s00449-012-0830-1.

Lee Chang, K.J., Dunstan, G.A., Abell, G.C., Clementson, L.A., Blackburn, S.I., Nichols, P.D., et al. (2012). Biodiscovery of new Australian thraustochytrids for production of biodiesel and long-chain omega-3 oils. Appl. Microbiol. Biotechnol. 93(5), 2215-2231. doi: 10.1007/s00253-011-3856-4.

Liang, Y., Sarkany, N., Cui, Y., Yesuf, J., Trushenski, J., and Blackburn, J.W. (2010). Use of sweet sorghum juice for lipid production by *Schizochytrium limacinum* SR21. Bioresour. Technol. 101(10), 3623-3627. doi: 10.1016/j.biortech.2009.12.087.

Nguyen, H.C., Su, C.-H., Yu, Y.-K., and Huong, D.T.M. (2018). Sugarcane bagasse as a novel carbon source for heterotrophic cultivation of oleaginous microalga *Schizochytrium* sp. Ind. Crops Prod. 121, 99-105. doi: 10.1016/j.indcrop.2018.05.005.

Thyagarajan, T., Puri, M., Vongsvivut, J., and Barrow, C.J. (2014). Evaluation of bread crumbs as a potential carbon source for the growth of *Thraustochytrid* species for oil and omega-3 production. Nutrients 6(5), 2104-2114. doi: 10.3390/nu6052104.

Wang, Q., Sen, B., Liu, X., He, Y., Xie, Y., and Wang, G. (2018). Enhanced saturated fatty acids accumulation in cultures of newly-isolated strains of Schizochytrium sp. and *Thraustochytriidae* sp. for large-scale biodiesel production. Sci. Total Environ. 631, 994-1004. doi: 10.1016/j.scitotenv.2018.03.078.

Yaodong Ning, X.L. (2020). RETRACTED: Enteromorpha hydrolysate as carbon source for fatty acids production of microalgae *Schizochytrium* sp. Energy 203, 117900. doi: 10.1016/j.energy.2020.117900.

Yin, F.W., Zhu, S.Y., Guo, D.S., Ren, L.J., Ji, X.J., Huang, H., et al. (2019). Development of a strategy for the production of docosahexaenoic acid by *Schizochytrium* sp. from cane molasses and algae-residue. Bioresour. Technol. 271, 118-124. doi: 10.1016/j.biortech.2018.09.114.

Yokochi, T., Honda, D., Higashihara, T., and Nakahara, T. (1998). Optimization of docosahexaenoic acid production by *Schizochytrium limacinum* SR21. Appl. Microbiol. Biotechnol. 49(1), 72-76. doi: 10.1007/s002530051139.

Yu, X.J., Liu, J.H., Sun, J., Zheng, J.-Y., Zhang, Y.-J., and Wang, Z. (2016). Docosahexaenoic acid production from the acidic hydrolysate of Jerusalem artichoke by an efficient sugar-utilizing *Aurantiochytrium* sp. YLH70. Ind. Crops Prod. 83, 372-378. doi: 10.1016/j.indcrop.2016.01.013.

Zhang, S., Chen, X., Sen, B., Bai, M., He, Y., and Wang, G. (2021). Exogenous antioxidants improve the accumulation of saturated and polyunsaturated fatty acids in *Schizochytrium sp.* PKU# Mn4. Mar. Drugs 19(10), 559. doi: 10.3390/md19100559.
